# PrEP Method Switching: Will it Yield Greater Coverage of HIV Protection? Applying Lessons Learned from Family Planning to Guide Future Research in the Context of PrEP Choice

**DOI:** 10.1007/s11904-024-00704-1

**Published:** 2024-07-24

**Authors:** Courtney McGuire, Margaret A. Atieno, Theresa Hoke, Patriciah Jeckonia, Kevin K’orimba, Lara Lorenzetti, Kenneth Ngure, Marie Merci Niyibeshaho, Njambi Njuguna, Kristine Torjesen, Virginia Fonner

**Affiliations:** 1 FHI 360, 359 Blackwell Street, Suite 200, Durham, NC 27701 USA; 2 LVCT Health, Nairobi, Kenya; 3 School of Public Health, Jomo Kenyatta University of Agriculture and Technology, Nairobi, Kenya; 4 Department of Global Health, University of Washington, Seattle, USA; 5 FHI 360, Nairobi, Kenya

**Keywords:** HIV Prevention, Pre-exposure prophylaxis, Method Choice, Method Switching, Method Discontinuation, Life course

## Abstract

**Purpose of Review:**

Despite the growing availability of oral PrEP, coverage remains suboptimal. Through the introduction of additional PrEP methods, including vaginal rings and long-acting injectable formulations, health systems globally are on the cusp of offering PrEP methods that vary by route of administration, efficacy, and frequency of use. With PrEP choice, it will be important to explore PrEP use patterns to better understand how the ability to choose and switch products affects coverage and continuation. In this review, we draw parallels with family planning (FP) by summarizing how method choice and product switching affected contraceptive coverage globally, synthesize what is known about PrEP product switching, and outline evidence gaps to help guide future research on PrEP switching in the context of choice.

**Recent Findings:**

Decades of research in FP has demonstrated that product switching is common and can lead to more satisfaction and increases in contraceptive use. While research on PrEP product switching is nascent, findings suggest switching is common, and that providing more than one PrEP option can increase coverage. Key evidence gaps include understanding product switching in the context of full versus constrained choice, switching in the context of temporary need, and developing interventions that promote product switching for those who could benefit.

**Summary:**

Providing choice and allowing people to start, stop, and switch products according to their needs and desires is a core component of a rights-based approach to HIV prevention. More research is needed to better understand what drives use patterns, including switching, and how to leverage choice to improve coverage. Standard definitions —some of which have been proposed in this review—are needed to inform comparable measurement. Finally, there is a need to holistically frame PrEP use to acknowledge changes in need over the life course, thus making method switching a standard part of HIV prevention.

## Introduction

Globally, rates of uptake and continuation on pre-exposure prophylaxis (PrEP) to prevent HIV remain suboptimal, with estimates suggesting that 41% of oral PrEP users discontinue within six months [1]. Considering these challenges, PrEP choice is poised to be a game changer for HIV prevention as end-users are offered the option to choose among HIV prevention products, including oral PrEP, the dapivirine vaginal ring (PrEP ring), long-acting injectable cabotegravir (CAB PrEP), condoms, and eventually, other products in the development pipeline. As choice increases, new patterns of use will emerge, such as transitioning on and off PrEP and switching between PrEP products, due to changing life circumstances, method dissatisfaction, lack of access to desired product, or personal interest and preference.

Because oral PrEP has been the only PrEP method available, interventions to support PrEP use have focused on product-specific uptake, adherence, and/or continuation. With emerging PrEP choices, there is a need to understand switching between PrEP methods and other transitions across varied HIV prevention options, and to develop interventions to support people through such changes. This review summarizes current evidence on PrEP method switching, inspired by key lessons drawn from experience with family planning (FP) programming, proposes definitions of key switching transitions (Table 1), and identifies evidence gaps that the HIV prevention research field should fill as PrEP method choice is introduced. While existing data on FP method switching focuses on cis-gender women, instances of PrEP method switching to-date have involved more diverse populations, including cis-gender and transgender populations. As HIV prevention is relevant to populations beyond cis-gender women, this review is inclusive of all populations who have experienced PrEP method switching given the important lessons for all that can be drawn from FP and PrEP switching experiences.

**Table 1:** Key definitions and measurement considerations for switching

Definitions
**HIV prevention transitions:** Encompasses all shifts from one HIV prevention method/strategy to another, including: switching between PrEP methods; switching from PrEP to a non-PrEP method, such as PEP or condom use, and vice versa; or cycling on and off PrEP, with either no HIV prevention method used in the interim periods, or use of a non-PrEP method.
**Within the umbrella of HIV prevention transitions, there are transitions specific to PrEP method switching:**
**PrEP method switching:** Stopping one PrEP method and choosing to use another PrEP method, either permanently or temporarily. Using oral PrEP as a bridge to cover prevention gaps in the context of a planned delay in a CAB PrEP injection is an example of a temporary switch.
**Continuous PrEP switching:** Switching methods sequentially with no gap or a gap of 30 days or less between use of different PrEP methods. Additional research on the length of the gap between PrEP methods is needed to help refine the definitional timeframe.
**Interrupted PrEP switching:** Switching between PrEP methods, interspersed with periods greater than 30 days of no PrEP use or use of a non-PrEP method before the new PrEP method is chosen.
Measurement considerations
**Continuation:** In the context of choice, it will be important to distinguish between HIV prevention continuation, PrEP continuation, and product-specific continuation. HIV prevention continuation is inclusive of the time in which someone is using any HIV prevention method, including PrEP and non-PrEP methods, while in need. PrEP continuation is specific to the time in which someone reports using any PrEP method, inclusive of PrEP method switching (assuming a continuous switch). Product-specific continuation refers to the time spent on a specific PrEP product and would thus be exclusive of switching or would measure time until the point of a PrEP method switch.
**Discontinuation:** As above, in the context of choice it will be important to distinguish between discontinuation of HIV prevention more generally (i.e., cessation of any sort of product or strategy to prevent HIV), PrEP discontinuation (i.e., cessation of all forms of PrEP), and method-specific discontinuation. Of note, given individuals’ evolving needs, it could be more appropriate to discuss gaps/interruptions in HIV prevention (inclusive of PrEP and non-PrEP methods) and gaps/interruptions in PrEP use instead of using the general term “discontinuation” to better capture the impermanence of HIV prevention method use.

## Drawing Lessons from Decades of Contraceptive Method Choice and Switching

### What Have We Learned from FP Regarding Switching Prevalence, Reasons for Switching, and Impact on Coverage and Use?

To inform the design of PrEP services that support method switching, lessons can be drawn from the decades of experience expanding contraceptive method choice. The early decades of FP were focused on reducing fertility rates and population growth [2]. However, Judith Bruce’s seminal quality-of-care framework shifted the focus to client-centered service delivery and acknowledged that switching was common and could satisfy clients’ needs [3]. In 2018, an updated quality-of-care framework made five major revisions, including to the element of follow-up, with “information on follow-up requirements and guidance on the possibility of switching the method, provider, or service outlet” [2]. This update acknowledged the importance of a client making their own decision on their contraceptive use to ensure desired reproductive goals. In addition, evidence suggests that as more contraceptive methods were offered, overall modern contraceptive prevalence increased [4]. Increased contraceptive options provide more ways to satisfy individuals’ and couples’ contraceptive needs and preferences and offer opportunities to switch methods when needed or wanted.

Women choose to discontinue their initial contraceptive method and switch to another method for many reasons, with method-specific reasons commonly cited. Frequently, side effects, including increased bleeding or changes to sexual experiences, are cited as the primary driver of method switching [5, 6]. Switching contraceptive methods to alleviate unwanted side effects minimizes discontinuation rates for women with continued contraceptive needs while enabling them to have more productive and healthy lifestyles [7].

Many other factors influence women’s decisions to discontinue one method and initiate a new one, including the method’s efficacy and availability as well as interpersonal dynamics. Changing contraceptive methods can present an opportunity to switch to a more effective method, including from traditional to modern methods [8, 9]. Switching methods can also reflect the relative ease of discontinuing the initial method as indicated by higher percentages of oral contraceptive users who switch to other methods [8]. In some cases, switching decisions may not be method specific but instead reflect interpersonal relationships and power dynamics. Studies have noted both the impact of partner opposition on method switching [10] and the absence of association between partner opposition and method switching [11]. Product availability can also influence switching decisions. Women may navigate method stockouts by switching to another method, commonly a less effective method such as condoms or traditional methods like withdrawal [12, 13]. This was particularly challenging for women covertly using contraceptive methods [13].

There are important parallels between a woman’s experience switching contraceptive methods and switching PrEP methods. As new PrEP methods are added, the HIV prevention field will need to recognize, validate, and respond to the reasons people discontinue PrEP methods and choose to start another [14]. Supporting individuals through these transitions will likely lead to increased satisfaction with and use of PrEP methods.

### What Have We Learned from FP Regarding Switching Over the Life Course?

A woman’s contraceptive journey, including method selection, discontinuation, and switching, is influenced and shaped by her lived experiences. A qualitative study in Kenya used a modified life history approach to understand the contraceptive journeys of experienced adolescent and young female contraceptive users. Findings emphasized the importance of life transitions such as pregnancy and childbirth and changes in relationship status on a woman’s subsequent contraceptive choices. Many respondents’ transitions from coitally dependent methods (condoms and emergency contraception [EC]) occurred after the birth of their first child, whereas subsequent transitions were driven by desires to avoid side effects and achieve longer windows of contraceptive protection [15]. A longitudinal qualitative study in Benin categorized both male and female respondents into six types of users that may or may not have achieved their contraceptive needs. Similar factors emerged as crucial influences on the respondents’ contraceptive journey including fears of side effects, relationship dynamics, and access to services [16].

Individuals’ sexual and reproductive health experiences change throughout their life course along with their contraceptive and HIV prevention needs. Research and service delivery programs should be attentive to how an individual’s PrEP method choice will change as they navigate life changes such as relationship changes, pregnancy and childbirth, and seasons of risk [17].

### What Have We Learned from FP Regarding How to Measure Switching?

The FP field has developed innovative ways to measure contraceptive switching that—while open to improvement—offer important lessons for PrEP switching. The Demographic and Health Survey (DHS) contraceptive calendar guides women through interview questions to retrospectively record every monthly instance of contraceptive method use during a five-year period. It remains the standard primary data source for contraceptive dynamics across many geographies. DHS defines switching as when a woman discontinues a method and switches to another contraceptive method in the following month or indicates that she wanted a more effective method and switched to another contraceptive method within two months of discontinuation [18]. Other studies measured switching through longitudinal surveys, cross-sectional surveys with women selected for interview based on their contraceptive decisions 12 months previously, and weekly text message-based surveys that inquired about women’s need and contraceptive use [6, 9, 10, 19–21]. The FP field has recognized the importance of measuring not only contraceptive method switching to address discontinuation but also the importance of counseling on availability of switching. Incorporated measurements of counseling on the availability of switching have demonstrated an association with contraceptive continuation [22]. The FP and PrEP fields both measure challenging subject matter, including private and sensitive topics such as sexual risk, unintended pregnancies, and HIV seroconversion. With the pressing need to develop and refine measures of PrEP switching, the HIV prevention field can continue to build upon lessons from years of contraceptive measurement [23].

The FP field has vast experience offering a variety of methods with different characteristics to match women’s needs. Quality of care is linked to improved client satisfaction, increased contraceptive knowledge, and contraceptive use. There are multiple measurement frameworks and approaches to evaluate quality of care with outstanding needs for standardized measurements [24–26]. Over the past decade, there has been increased attention to ensure voluntary, rights-based FP [27] through frameworks [28], interventions [29] and measurement [30].

Through contraceptive choice, women have long made decisions to switch methods based on their reproductive needs and desires. These sexually active women of reproductive age are often also at risk of HIV infection. As new prevention methods become available, the HIV field can continue a tradition of identifying lessons learned from FP and adapting them to support and achieve HIV prevention goals [31–33].

## Limited PrEP Method Choice and Data on Switching to Date

### Overview: Expansion in HIV Prevention Methods

Oral PrEP changed the HIV prevention landscape by offering an effective antiretroviral drug option. In 2015, the World Health Organization (WHO) recommended that people at substantial risk of HIV infection be offered once-daily tenofovir disoproxil fumarate (TDF)-based oral PrEP as an option for HIV prevention as part of combination approaches. As of 2021, two-thirds of countries globally have adopted the WHO PrEP recommendations [34], resulting in increased PrEP usage globally. At the end of 2023, 6,220,507 PrEP initiations had occurred globally, with almost all representing oral PrEP usage [35].

While the introduction of daily oral PrEP was pivotal, for years the only other PrEP option was a different dosing schedule: event-driven PrEP (ED-PrEP) for certain populations. WHO added ED-PrEP to its guidelines for men who have sex with men (MSM) in 2019 and expanded the guidance in 2022 to include all cisgender men [36], recommending the use of a double dose of oral PrEP between two and 24 h before sex, a third pill 24 h after the first two pills, and a fourth pill 48 h after the first two pills. This 2–1-1 dosing schedule is appropriate for men who have infrequent sex and can plan for sex at least two hours in advance. ED-PrEP is not yet approved for use by women [37]. Additionally, another formulation of oral daily PrEP, tenofovir alafenamide with emtricitabine (TAF/FTC), has been shown to be as effective as TDF-FTC among MSM and transgender women who have sex with men [38, 39]. While several studies have begun to explore outcomes among those who switch from TDF/FTC to TAF/FTC [40, 41] we did not include studies where switching was limited to the same PrEP modality but only different drug regimens, such as F/TDF and F/TAF. While switching formulations will become more important as PrEP options expand, this review focuses on the experiences of switching between different modalities and schedules.

The PrEP ring was recommended by WHO in early 2021 as an additional prevention choice for cisgender women at substantial risk of HIV infection from vaginal sex [42]. The PrEP ring can be worn inside the vagina continuously for one month before being replaced, offering women a discreet form of HIV prevention. It is a female-initiated method that offers women a “set it and forget it” prevention option.

CAB PrEP was recommended by WHO in mid-2022 [43]. CAB PrEP is delivered via intramuscular injection, with the first two injections administered one month apart, followed by injections every two months. CAB PrEP’s injection modality offers users additional discretion and flexibility. Notably, CAB PrEP has a relatively long pharmacokinetic tail, with the drug persisting in the body for up to one year following cessation but not at sufficient levels to prevent HIV [44]. This has generated heightened concern for potential drug resistance if HIV is acquired during the tail period [45], leading to clinical recommendations to switch to other effective HIV prevention methods, such as condoms or oral PrEP, following CAB PrEP discontinuation.

### Evidence on PrEP Switching: Oral PrEP and Event-Driven PrEP

Much data on switching PrEP modalities comes from research among MSM in high-income countries that involved switching between daily oral PrEP and ED-PrEP. In general, results suggest that men switched methods use schedules to match their evolving sexual and lifestyle needs.

We reviewed five studies examining switching between taking oral PrEP daily or based on events (ED-PrEP) among MSM. These studies were conducted in Belgium, the Netherlands, Taiwan, Burkina Faso, Côte d’Ivoire, Mali, and Togo. Among these studies, from 13 to 34% of participants switched PrEP regimens within the study’s timeframe, with most studies having about one in five participants switch regimens.

The most commonly observed switch was from daily oral PrEP to ED-PrEP [46–48]. In a multi-country study in west Africa, 42% of participants switched from oral PrEP to ED-PrEP as opposed to 13% who switched from ED-PrEP to oral PrEP [46]. Participants who switched to ED-PrEP were motivated by a change in sexual relationships, including having less sex, or a desire to take fewer pills [49, 50]. Challenges with the daily dosing regimen were reflected in a study in Taiwan where those who switched to ED-PrEP were less likely to have taken daily PrEP correctly compared to those who did not switch from oral PrEP [51]. Additionally, among participants in Belgium who discontinued PrEP, the probability of re-starting with ED-PrEP was 35% compared to 13% for daily oral PrEP [48], suggesting a lower barrier to use for ED-PrEP.

In a study in Amsterdam, switches from ED-PrEP to oral PrEP were more than twice as likely to occur compared to the reverse. Within this study population, median time until first switch to ED-PrEP among daily oral PrEP users was 1.1 years, compared to 0.5 years among ED-PrEP users who switched to daily oral PrEP [52]. PrEP users who switch from ED-PrEP to oral PrEP commonly cite difficulties and stress with remembering the on-demand dosing schedule, with some finding the oral PrEP regime more convenient [49, 52]. Additionally, participants discussed switching to daily PrEP due to changes in sexual relationships, including increased number of sexual partners [49, 52].

While ED-PrEP is not yet approved for women [53] and these studies are mainly conducted in high-income settings, they provide important methodological and programmatic considerations for implementing PrEP choice. Most notably, these studies have demonstrated how to measure product choices over longer time periods and include qualitative methods to examine the complexities of PrEP method switching.

### Evidence on PrEP Switching: Oral PrEP and PEP

Switching between PrEP and post-exposure prophylaxis (PEP) offers another opportunity to examine HIV prevention method decisions involving switching. The Sustainable East Africa Research in Community Health (SEARCH) SAPPHIRE study is a cluster randomized trial in Uganda and Kenya with a dynamic choice model for HIV prevention. The study offered choice of HIV prevention product (PrEP or PEP), service location (clinic or home visits), and HIV testing modality. Throughout three SAPPHIRE studies in different service delivery locations (antenatal care, outpatient departments, and via community health worker), a greater proportion of participants used oral PrEP compared to those in control communities [54–56]. Within the community health worker study, selection of PrEP increased from 40 to 48% during the study, while PEP selection declined from 46 to 24% [55]. In the other service delivery locations, only 10–15% of participants ever chose PEP [54, 56]. The dynamic choice model resulted in large increases in biomedical coverage for intervention participants, with 70% of follow-up time for antenatal care intervention participants biomedically covered compared to 29% for control participants [56].

### Evidence on PrEP Switching: Oral PrEP and CAB PrEP

Limited evidence exists on individuals’ switching decisions that includes CAB PrEP as an option. Open-label extension periods offer a first glance at how PrEP users respond to method choice that includes CAB PrEP. In HIV Prevention Trials Network (HPTN) 083 and HPTN 084’s open-label extension, most participants chose CAB-PrEP [57, 58] when offered a choice between CAB PrEP and oral PrEP. In both studies, the most common reason for method selection was a preference for the way the method was delivered or a dislike of how the other method was delivered. In HPTN 084, product choice varied by study country, with 92% of study participants in Botswana selecting CAB PrEP compared to 69% in Uganda. Overall, participants in HPTN 084 expressed very little decisional conflict when making their open-label extension method choice, with CAB PrEP users having a stronger perception they made a method choice that they would stick to compared to oral PrEP users [58]. These findings highlight the strong interest in PrEP methods with different delivery characteristics and experienced users’ confidence in their ability to make their method choice.

The SEARCH SAPPHIRE study recently expanded to include CAB PrEP in addition to oral PrEP and PEP. With the addition of CAB PrEP, interim results suggest that coverage among intervention participants increased to 70%, compared to 13% in the standard-of-care arm. Notably, one in four participants used two different methods (among oral PrEP, CAB-PrEP, or PEP) over the course of the study, reflecting participants’ ability to change methods to meet their evolving needs [59]. PrEP ring will be added as an additional PrEP method in Phase III of the SAPPHIRE study, offering initial evidence on switching between multiple PrEP methods.

### Evidence on PrEP Switching: Oral PrEP and PrEP Ring

The PrEP ring has been investigated in only a few studies outside of clinical trials. The REACH study in South Africa, Zimbabwe, and Uganda sought to assess choice of and adherence to PrEP ring and oral PrEP among adolescent girls and young women (AGYW) ages 16–21 years. Young women were randomized to either method for the first two phases and then allowed to choose oral PrEP, PrEP ring, or neither during the final six months. During the choice period, 67% of participants chose PrEP ring, while 31% chose oral PrEP [60]. Method adherence remained relatively high during the choice period. Correlates of high adherence varied between methods, suggesting the person-centered benefits of offering method choice [61]. While not in a real-world setting, these results offer an example of choice and potential switching patterns among young women when offered two biomedical prevention options.

### Discrete Choice Experiments and Hypothetical Studies

While research on PrEP method switching is limited, a body of relevant research exists that will prove useful as PrEP choice becomes more available: discrete choice experiments (DCEs). DCEs present respondents with a choice between two or more hypothetical options (or “sets”) to examine their preferences and trade-offs between attributes related to PrEP products or scenarios. Wulandari and colleagues conducted a systematic review of DCEs studying PrEP choices. As potential PrEP users were offered different types of PrEP, they identified valued attributes in their decision-making including product cost, perceived effectiveness, dosing frequency, confidentiality, and service delivery model [62].

Clinical trials using placebo products to assess accessibility and adherence offer key insights into potential PrEP method preferences and use patterns, including switching. Young female participants in the Quatro Clinical Crossover Study in South Africa and Zimbabwe were asked to use a pre-coitally inserted film, vaginal tablet, vaginal gel, and monthly ring for one month each. They could choose their preferred method for the fifth and final month of the study. Participants’ method preferences varied across countries and settings, reflecting the critical need for a variety of PrEP method options to satisfy differing user needs. Additionally, participants’ preferences shifted after they learned more about a method and gained experience using it [63]. In the Tablets, Ring, Injections as Options (TRIO) Study, young women in South Africa and Kenya were randomized to use each placebo delivery method (ring, injection, pill) for one month before being able to select a final method to use for the last two months. Injections were the most preferred and had the best compliance in the final stage [64]. The UChoose Study used contraceptive options as a proxy for HIV prevention products to better understand young South African women’s preferences for product attributes of ring, pills, and injectables. The study found that injection users were significantly more likely to feel that their method was convenient to use compared to ring users. Additionally, more ring participants requested to switch to another contraceptive option compared to injection users [65]. Hypothetical studies offer valuable information on the nascent field of PrEP switching but are not able to provide critical information on how side effects from an active product would influence switching, a key factor for contraceptive switching.

### Evidence Gaps

Given that PrEP choice is only now being introduced—mostly in the context of implementation research but also through programmatic rollout in select countries—there are substantial gaps in our understanding of PrEP method switching.

#### We Need to Understand Switching in the Context of Both Full and Constrained Choice, Including Implications for Uptake, Continuation, and Coverage for Each Scenario

Many ongoing and planned implementation studies are offering or will offer PrEP choice [66, 67]. This body of research will provide critical data on the frequency of product switching in the context of full choice, reasons for switching, and the impact of choice on uptake, continuation, and HIV prevention coverage.

Additionally, some implementation studies are including populations who might not be medically eligible for all available PrEP methods, such as pregnant and/or breastfeeding people (PBFP). These studies will shed light on how people make decisions when choices are constrained and potential implications when one is unable to receive one’s method of choice. PrEP implementation among PBFP often has not been prioritized despite known elevated risks of HIV during pregnancy [68]. Training providers to offer choice and allowing women to switch between PrEP methods given their pregnancy and/or breastfeeding status could help meet and support the unique needs of these populations.

Other circumstances in which PrEP choices could be constrained include product stockouts or stockouts of relevant commodities, including HIV test kits, and limited availability of certain methods based on clinic, provider (i.e., some providers might only be trained on certain methods), population, or delivery settings (i.e., delivery via community-based mechanisms might be challenging for certain methods). Stockouts of both drug and HIV test kits have been recognized as barriers to PrEP continuation among programs delivering oral PrEP [69, 70]. Regarding CAB PrEP, there is concern that stockouts could lead to forced product switching to cover prevention gaps. If the product switched to is less desired, or if adherence cannot be maintained, product discontinuation could put the individual at risk for drug resistance if HIV is acquired during the CAB PrEP tail period. Additionally, a recent consultation with PrEP providers revealed concerns that injectable PrEP formulations might re-medicalize PrEP, thus reducing gains in offering differentiated service delivery [71].

FP provides a successful model for delivering products outside of clinic settings, and this is a critical avenue for increased availability and choice. It is equally important for PrEP programs to ensure availability of a range of products in clinics and community-based settings. Several ongoing studies are testing the feasibility of different service delivery models for offering PrEP choice, including through facility and community-based sites [67]. Results will yield important information about differentiated service delivery in the context of PrEP choice, including switching patterns across delivery approaches. Ensuring PrEP choice is integrated into existing services, including FP services, could also help ensure choice. As health care providers, community clinics, and peers are among the most trusted sources of information, it is important that PrEP is available through these sources and that they receive accurate and comprehensive information on PrEP method choice. It is equally important that community-based demand generation and PrEP campaigns are inclusive of PrEP choice and the possibility of switching methods.

#### More Evidence is Needed to Inform Guidance on How to Switch PrEP Methods from a PrEP to a Non-PrEP Method (and Vice Versa)

In 2022, WHO released guidance on differentiated and simplified service delivery for oral PrEP, including daily and ED-PrEP [36]. However, as the guidelines pertain to oral PrEP, there is no mention of how to switch PrEP methods or use intermittently. More guidance will be needed on these topics, including whether product overlap is recommended during switching and at what point a client is protected by the new method following a switch. Guidance will be especially relevant for CAB PrEP as it imposes a return-to-clinic burden that might require flexibility. For example, if a person is away from home during their next scheduled injection, they might need to switch temporarily to oral PrEP—known as “bridging”—until they can resume/restart injections [72]. Bridging with another effective product during the tail period is also recommended. However, guidelines are needed on how to effectively implement bridging.

#### There are Other Considerations for Measuring and Monitoring Switching in the Context of PrEP Choice

Current global PrEP indicators that measure collective use do not account for product switching [73]. Additionally, common definitions are lacking for many aspects of PrEP use, including discontinuation, intermittent use, and event-driven use [73]. Many of these issues have been outlined by Dunbar et al. [74], although new measurement challenges have arisen due to PrEP choice, including lack of switching definitions and measures. For example, in the context of choice and recognizing that HIV prevention journeys often follow non-linear trajectories, it is unclear whether using terms like “discontinuation” are useful, or whether it would be more appropriate to talk about gaps in PrEP use, or gaps in HIV prevention more generally. Conversely, it will also be important to ensure that definitions of continuation are inclusive of PrEP method switching. We have outlined key definitions and measurement considerations in Table 1, although more conceptualization and operationalization of these definitions are needed. Understanding and monitoring “typical” patterns of switching also could help identify potential issues (e.g., dramatic increases or decreases in switching could signal stock shortages, provider biases, or other constraints that warrant intervention). Finally, measures of quality of PrEP choice counseling are lacking. Some measures on quality of counseling in FP, such as the Methods Information Index Plus, include whether clients were told about the possibility of switching as a marker of quality [22]. Quality measures adapted for PrEP choice counseling should similarly include questions relevant to switching.

#### We Need Evidence-Based Interventions that Support PrEP Continuation in the Context of PrEP Choice, Including those that Allow and Support Product Switching

**Fig. 1:**
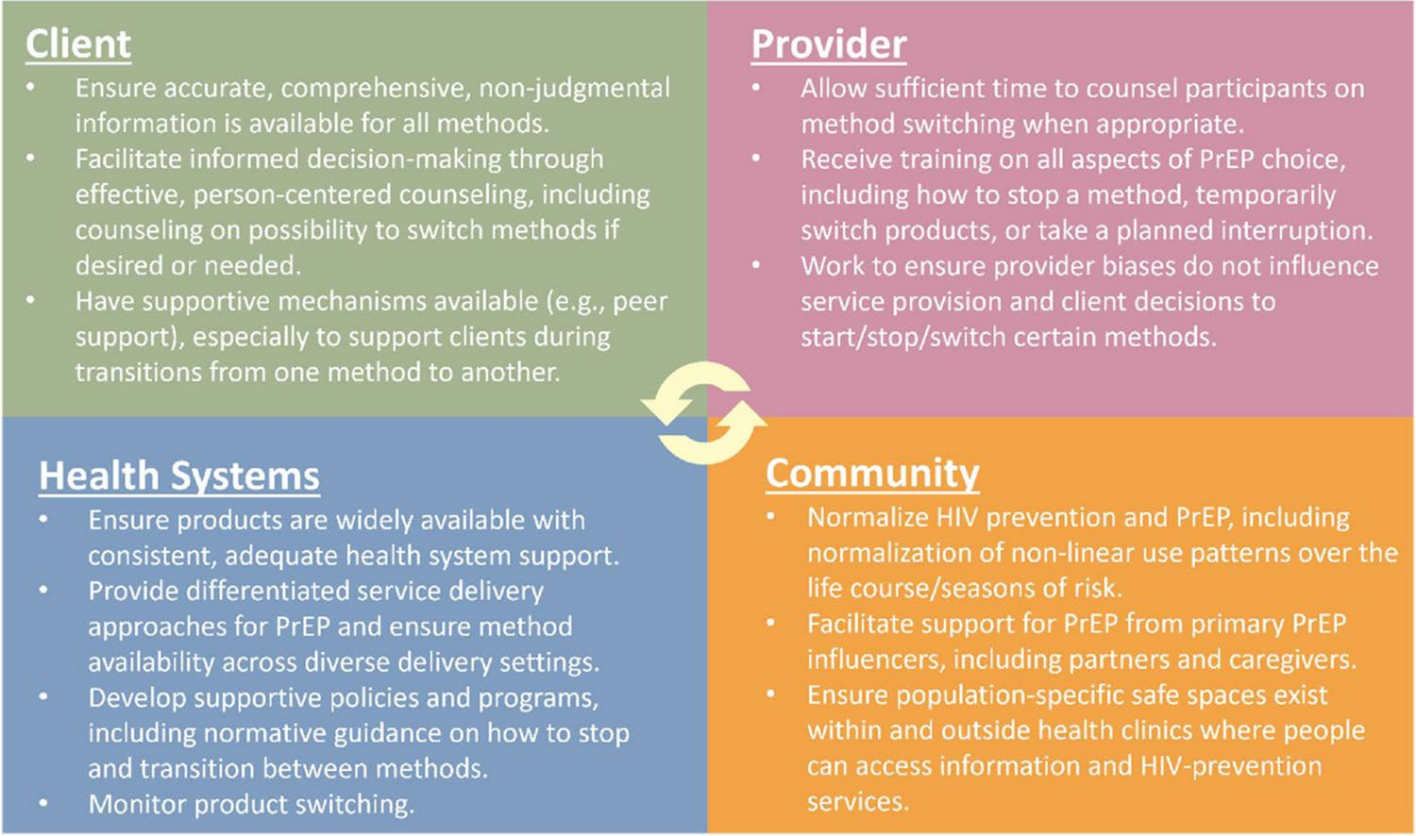
Potential mechanisms to acknowledge and support PrEP method switching in the context of PrEP choice

No interventions currently exist to support switching between PrEP products or transitioning between PrEP and non-PrEP HIV prevention methods. Evidence-informed service delivery norms are needed to facilitate PrEP choice, such as by ensuring that clients are fully aware that method switching is possible if one method proves undesirable or intolerable, and that people are counseled on alternative choices in cases where method choice becomes constrained. Figure 1 presents mechanisms through which method switching could be permitted and supported. Conceptualizing switching, and interventions to support switching, can also be broadened to consider transitions from PrEP to non-PrEP methods (and vice versa), such as PEP or condom use. These transitions become increasingly relevant when considering changing HIV prevention needs over the life course. This broader conceptualization aligns with more recent, nuanced definitions of “prevention effective use,” or use of PrEP only during periods of risk [75].

#### Considerations are Needed for Future Product Introduction, Including How to Ensure People are Fully Informed about New Methods as they Come on the Market and Can Access their Preferred Method

Evidence presented here primarily comprises studies on oral PrEP, PrEP ring, CAB PrEP, and PEP. However, more HIV prevention products are in development. The addition of each new product will reshape the market and will require health system efforts to ensure product availability, provider training, and demand generation. Programs will need to plan carefully for these additions to ensure clients are fully informed of their expanding options. Ensuring post-trial access for products following clinical trials and subsequent open-label extension studies and demonstration projects will require collective efforts from researchers, product manufacturers, program implementors, and governments.

## Conclusion

Understanding how to enable PrEP choice and support method switching is not just a pathway to improving PrEP continuation, but an imperative that community members are demanding. In September 2023, the African Women’s HIV Prevention Community Accountability Board released its HIV Prevention Choice Manifesto, signed by the UNAIDS Executive Director, calling for political and financial commitments to support HIV prevention choice [76]. Policymakers, researchers, advocates, and end-users alike acknowledge the importance of PrEP choice, but require tools and resources to ensure a true choice is available to all. The importance of offering and allowing choice is also conveyed in the Choice Principles, which employ a human rights-based approach and specify that HIV prevention markets must be grounded in informed choice to accommodate users’ dynamic needs and preferences [77]. There are clear parallels to promoting HIV prevention choice—and switching as a core component of choice—and the rights-based approach to FP that recognizes rights related to receiving access to services inclusive of choice [78].

Offering of PrEP choice is just beginning, and as demonstrated through the limited evidence identified in this review, more research is needed to understand product switching and whether offering PrEP choice inclusive of switching will lead to better HIV prevention coverage. More specifically, research is needed to inform how to measure PrEP method switching, reasons for switching, patterns of use following switching, as well as interventions to support PrEP method switch and broader HIV prevention transitions. Health systems research is needed to find solutions to persistent supply chain, human resource, and information management problems impeding effective delivery of even single PrEP methods. Additionally, development of quality PrEP choice counseling measurement tools should include items related to being informed that switching methods is possible. These efforts will ensure a better understanding of switching and its role in ensuring people's ability to fulfill their HIV prevention needs over the life course.
